# Can 28-Month-Old Children Learn Spatial Prepositions Robustly from Pictures? Yes, When Narrative Input Is Provided

**DOI:** 10.3389/fpsyg.2016.00961

**Published:** 2016-07-15

**Authors:** Katharina J. Rohlfing, Kerstin Nachtigäller

**Affiliations:** ^1^Department of Arts and Humanities, Paderborn UniversityPaderborn, Germany; ^2^Bielefelder Institut für frühkindliche Entwicklung e.V.Gütersloh, Germany

**Keywords:** language acquisition, spatial prepositions, slow mapping, cognitive linguistics, book reading

## Abstract

The learning of spatial prepositions is assumed to be based on experience in space. In a slow mapping study, we investigated whether 31 German 28-month-old children could robustly learn the German spatial prepositions *hinter* [behind] and *neben* [next to] from pictures, and whether a narrative input can compensate for a lack of immediate experience in space. One group of children received pictures with a narrative input as a training to understand spatial prepositions. In two further groups, we controlled (a) for the narrative input by providing unconnected speech during the training and (b) for the learning material by training the children on toys rather than pictures. We assessed children’s understanding of spatial prepositions at three different time points: pretest, immediate test, and delayed posttest. Results showed improved word retention in children from the narrative but not the control group receiving unconnected speech. Neither of the trained groups succeeded in generalization to novel referents. Finally, all groups were instructed to deal with untrained material in the test to investigate the robustness of learning across tasks. None of the groups succeeded in this task transfer.

## Introduction

When acquiring language, children make gains in their word knowledge. Depending on the word class, the word knowledge is different. In this article, we will study prepositions. These belong to the closed class, meaning that they are limited in number and mark relationships between objects or persons. For many researchers, prepositions are acquired in different ways compared to nouns. Support for this claim comes from cross-linguistic studies revealing a specific acquisition order reflecting how children’s universal experience with space guides their cognitive development (Johnston and Slobin, 1979; Johnston, 1984, 1988; Sinha et al., 1999). It is thus assumed that the word knowledge of spatial prepositions involves knowledge about space (Johnston and Slobin, 1979; Landau, 1994; Bowerman, 1996). Landau (1994) reports experiments showing that 3-year-old children attended to different properties in two conditions, in which they learned either nouns or prepositions: In the noun condition, they attended to an object’s shape, in the preposition condition, to its position. Clearly, at 3 years, children can use syntactic properties to converge on the correct meaning category for a novel word. Nonetheless, a “direct pipeline” from spatial words to underlying non-linguistic spatial concepts cannot be assumed (Bowerman, 1996, p. 416). Although it has been shown that an increase in acting out spatial relationships leads to a better understanding of spatial prepositions in children (e.g., Rohlfing, 2006), no previous study has examined whether children can gain in spatial knowledge from pictures. Surely, pictures cannot provide direct experience with space. However, they evoke narratives (Reese et al., 2010; Nachtigäller and Rohlfing, 2011a), in which rich semantic input might facilitate the acquisition of spatial prepositions. A further question is whether children who have learned meanings about spatial prepositions from pictures can transfer this knowledge to other contexts than pictures. This is an important aspect of learning, because we know that word learning is a prolonged process extending beyond the initial exposure to a new word (McGregor, 2004; Wojcik, 2013). Hence, in this article, we address three aspects of learning from pictures: we test the benefit of (a) narratives for learning spatial prepositions, (b) the benefit of the application of narratives with pictures, and (c) whether the meaning of a word acquired in this semantic context can be transferred to other situations.

### Benefits of Narratives

Narratives place a new word into a context. This context is likely to support the development of semantic networks in young children. Such a network is a means of organizing relationships between words and any other kinds of experience (Binder and Desai, 2011); and these relationships are important for word semantics (Capone Singleton, 2012). One function of such a semantic network is to support additional word learning via inference (Wojcik and Saffran, 2013). Hoff and Naigles (2002) argue that a child’s interpretation of a less familiar word will be enhanced when it is framed by more familiar words (see also Goodman et al., 1998; Tomasello, 2003). Moreover, in a recent study, Horowitz and Frank (2015) have shown that it is not just individual words that help children to disambiguate a reference but the coherence of the words as well. Hence, children can infer a meaning not only because of what they know about the relationships between words (Wojcik and Saffran, 2013) but also because the input can constrain the inference by providing a specific linguistic structure. We will call this kind of input that emphasizes relationships between words the “semantic context” (cf. Goodman et al., 1998 when focusing on a lexical contrast as one example of a relationship between words). Rich semantic context occurs typically in book reading in which narratives can be broadly defined as a sequentially *organized* representation of *event* units. This organization can be characterized by (a) the linguistic devices that tie clauses cohesively together and (b) the causal and temporal coherence of the units that brings them into a relationship (Bamberg, 2012).

Causality is a central property of narrative structures (Labov and Waletzky, 1973; Mandler and Johnson, 1977). Booth (2009) investigated the initial comprehension and retention of words for new objects in 3-year-old children after exposure to either an object’s causal properties (e.g., “these are used to grind up food,” p. 1244) or its non-causal properties (e.g., “these have a part inside that is made of gold,” p. 1244). Results revealed that children’s retention performance was better when new words were trained with causal descriptions. Importantly, the differences between conditions did not become apparent when tested immediately after training, but only after a delay of several days. The author concluded that when 3-year-old children are provided with causal information, they may need a period of consolidation and/or recovery from training to increase the likelihood of a label being learned (Booth, 2009).

Similarly, in a previous study (Nachtigäller et al., 2013), we demonstrated that providing a structured semantic context in which a new word occurred facilitated young children’s vocabulary learning. More specifically, we used toys to train 21-month-old children to understand the spatial preposition *under* with toys: in one group, the preposition *under* was embedded into a narrative context with semantically coherent sentences that were temporally and causally related. For example, the children heard stories such as “It is raining. The man does not want to get wet. He goes *under* the roof.” The control group, in contrast, was exposed to a similar number of sentences and words but without the internal coherence of the sentences. They heard sentences such as “The man is small. He goes here. He is *under* the roof (Supplementary Material).” Results showed that providing a rich semantic context in the form of a narrative was beneficial for learning (Nachtigäller et al., 2013).

What mechanism underlies the beneficial effects of narrative structures on learning in both of these above-mentioned studies? We think that both studies can provide crucial support for the learnability of spatial prepositions from picture books without having to actually perform an act in space. There are three plausible explanations for this support:

Mandler’s (1984) approach focuses on narratives as a means of reducing cognitive load. The core of her approach is to organize individual units within a narrative characterized by linguistic cohesiveness and cognitive (temporal and causal) coherence. Each narrative thus delivers a hierarchical and ordered organization (called a story schema). These characteristics of the story schemas account for the beneficial effect on encoding and retrieval (Mandler, 1984). Thus, in the above-mentioned studies (Booth, 2009; Nachtigäller et al., 2013), the novel words were put within a predictable story schema making them more perceivable and easier to integrate into a meaning structure about an incident. This approach can be complemented by current embodied theories (Sadoski and Paivio, 2004; Barsalou, 2008), rendering meaning as a product of a schema that is composed of the activation (or simulation) of both the non-verbal coherence of the event units and the verbal representation. The benefits of narrative structures for learning can thus be explained by the mental representation of a new word being joined by a stronger memory trace left by the action information about the event. This can potentially enrich the child’s vocabulary knowledge with world knowledge.

In developmental studies, the embodied approaches to language have been barely considered in explanations of the positive effects that narrative elements in conversational style have on children’s memory development (e.g., Reese et al., 1993; Tessler and Nelson, 1994; Bauer and Wewerka, 1995; Boland et al., 2003). The application of the existing theories to developmental processes is also difficult, because in learning, it is necessary to consider not only the effect of the specific input in general but also, and more importantly, the effect of the specific input on the individual learner. In this vein, various studies have shown that narratives possess the potential to promote language learning, but that children’s existing language skills bolster these effects (Bauer and Wewerka, 1995; Boland et al., 2003; Nachtigäller et al., 2013). In fact, our study of 21-month-olds (Nachtigäller et al., 2013) revealed that those who were reported to be more advanced in their vocabulary development than their peers benefited more from the semantic context embedding a word within a causal and temporal event organization. In addition, findings by Gershkoff-Stowe (2002) as well as Arias-Trejo and Plunkett (2009) suggest that older children show a stronger bias toward semantically related words than younger children. With this in mind, in our study, we tested older children than in the previous study by Nachtigäller et al. (2013). We focused on children around the age of 28 months because Gershkoff-Stowe (2002) reported that children of this age showed a stronger bias toward semantically related errors in object naming than younger children. Our investigation focused on the prepositions *behind* and *next to* because these are both acquired at the age of 2–3 years, after children have already mastered *in, on*, and *under* (Johnston and Slobin, 1979). We hypothesized that children in the narrative condition would show better learning effects than children in a control group, because the narrative structure provides the children with a better simulation of an event that is needed to access the word meaning of a spatial preposition. We expected that in the narrative group, the general support of the system of meanings would be reflected in children’s ability to retain (see results in Booth, 2009) and generalize (see results in Rohlfing, 2006) the novel objects over the long-term. Our assumption is motivated by Capone Singleton’s (2012) view of word semantics. She suggests that the retrieval of a word (and its meaning) is influenced by how richly semantic information has been encoded. In addition, in line with embodied theories positing that meaning is presented as being both constrained and elaborated by the set of verbal associates that are activated (Sadoski and Paivio, 2004), we hypothesized that children with larger overall vocabularies would benefit more from narrative input that activates “verbal associates” (Gershkoff-Stowe and Hahn, 2007).

### The (Long-term) Benefits of Learning from Pictures

In their extensive review, Ganea and Canfield (2015) provide evidence that children as young as 15 months are not just able to pick up nouns from a pictorial representation of the objects. They can also generalize object properties to real objects. Thus, there is evidence suggesting that some word classes can be learned from a two-dimensional presentation, even though prepositions have not been studied in this way yet. There are several reasons for these effects, that all converge to the idea that pictures might be a special form of material promoting early learning. Firstly, Gelman et al. (2005) have shown that when mothers and their 34-month-old children jointly looked at pictures (in contrast to jointly playing with objects), they produced a higher proportion of generic and ostensive labeling phrases that might promote children’s generalizable knowledge about kinds of objects. Consequently, children might be more apt to apply the cognitive processes of generalization in this situation. Secondly, events in picture books are accessible in general, and other persons (not only adults but also siblings) can refer to exactly the same event (Nachtigäller and Rohlfing, 2011b, p. 135). Thus, joint picture book reading makes an event repeatable. Children benefit from repetitions (Horst et al., 2011) and “often demand to re-read” a particular book, thereby creating the recurrences (Snow and Goldfield, 1983, p. 553).

For our investigation, we thus hypothesized that the pragmatics of the situation (i.e., the fact that an event in a book can be re-read) would leverage the benefits of book reading for language learning in comparison to another situation such as free play. We predicted that children who have been trained with a narrative structure would demonstrate the ability to transfer their word knowledge to a different task irrespective of context, because their semantic memories have been strengthened by the stories. The learning content—spatial prepositions—is central to our investigation: whereas studies focusing on nouns (Ganea et al., 2008) can draw on the fact that selecting a picture and a real object on a tray can be achieved by the same pragmatic action of pointing to the referent, a child learning a spatial preposition has to perform different actions to reveal comprehension: In a book reading situation, a child *points* to a semantically matching picture; in a free play situation, in contrast, a child must *arrange* one object in a spatial relation to another.

## Materials and Methods

### Participants

Thirty-six German-learning children participated in this study. They were matched for age and gender prior to the beginning of the study to form comparable narrative and control groups. One group of 16 children (age *M* = 27.94 months, *SD* = 0.77) received input consisting of narrative structure (narrative group); a first control group of 15 children (age *M* = 28.0 months, *SD* = 0.76) received temporally and causally unconnected speech in the control condition (unconnected speech group). A second control group of 15 children (*M* = 27.93, *SD* = 0.70 years) received narrative input but was trained with objects (object group). Additional thirteen children were excluded due to fussiness or experimental dropout (e.g., illness). The groups did not differ in age, *F*(2,43) = 0.04, *p* = 0.96, gender, χ^2^(2, *N* = 46) = 0.38, *p* = 0.83, maternal education status, χ^2^(2, *N* = 46) = 0.25, *p* = 0.88, maternal time spent with child per day, χ^2^(2, *N* = 46) = 2.58, *p* = 0.28, or older siblings, *χ*^2^(2, *N* = 46) = 2.71, *p* = 0.26.

Monolingual language development was an inclusion criterion; children were excluded if they had atypical speech and hearing development, premature birth, or were twins.

During the training, we taught children the spatial prepositions *behind* and *next to*. Our decision led to the possibility that the 28-month-old children could well have heard the target prepositions before, but would probably have not yet built a complete lexical representation of them (Johnston and Slobin, 1979; Johnston, 1984, 1988). To control for their already existing knowledge of these prepositions upon enrollment in the study, we asked parents about their children’s comprehension and production of *behind* and *next to*. We did not exclude children who were reported as being able to produce and/or comprehend the target spatial terms because we were interested in determining whether a narrative presentation can aid them across various points in the word learning process. However, we did control for this individual variation by comparing the distribution of children who produced and/or comprehended the target prepositions in all conditions: children in the three experimental conditions did not differ in their comprehension of the spatial preposition *hinter* [behind], χ^2^(2, *N* = 45) = 0.05, *p* = 0.98) or in their production of this preposition, χ^2^(2, *N* = 45) = 1.59, *p* = 0.45). The same was true for the comprehension of the preposition *neben* [next to], χ^2^(2, *N* = 45) = 4.40, *p* = 0.11 as well as its production, χ^2^(2, *N* = 45) = 1.65, *p* = 0.44.

### Stimuli

#### Spatial Prepositions

Children’s understanding of these terms seems to change during the preschool years: early uses of *behind* and *in front of* typically occur with referent objects with intrinsic fronts and backs and includes canonical configurations (i.e., use of objects in their most common function), whereas later uses typically occur in the context of featureless reference objects and depend upon the speaker’s and listener’s point of view (Johnston and Slobin, 1979; Johnston, 1984, 1988). The meaning of the locative *behind* includes aspects such as accessibility, visibility, and proximity (Johnston, 1984). *Next to* limits the proximity character because it implies a closure. For the design of the relations, we thus regarded the trajector object with reference to the other present landmark objects that were present.

#### Training Items

**FIGURE 1:**
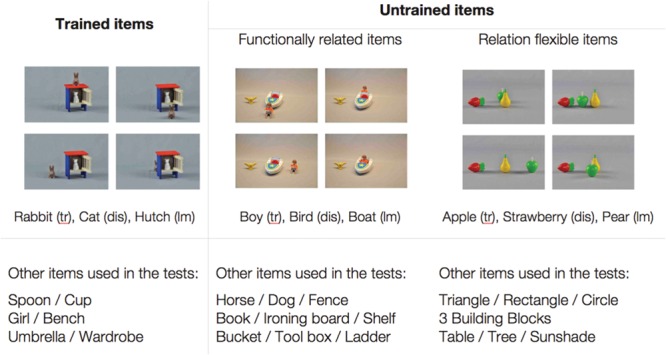
**Examples of items of each set category; tr: trajector object; lm: landmark object; dis: distractor object**.

A total of eight training items were applied: four for each trained target spatial relation (*behind* and *next to*). The training items consisted of one photograph of objects depicting the target spatial relationship arranged on a standard (A4) sheet of paper. These relationships were not canonical in the sense that children considered other spatial relations (e.g., *on* the bench) to be more appropriate here. **Figure 1** presents examples of training items for each target spatial relation.

**FIGURE 2:**
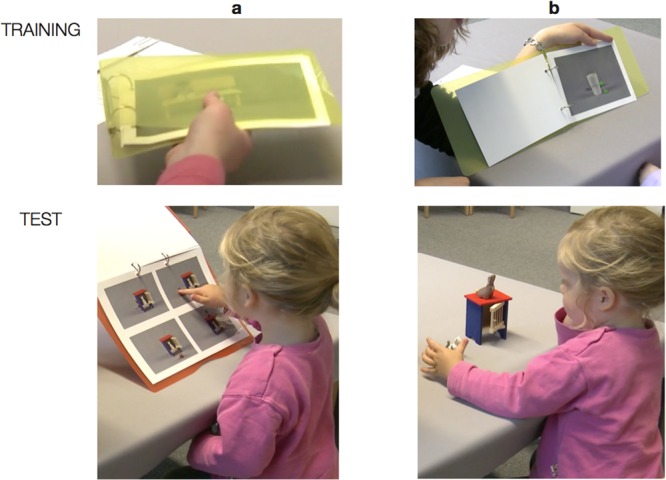
**Top:** Material used in the training (a and b show a yellow folder containing the eight training items. **Bottom**: In the test, children had to transfer the meaning of a preposition from one learning context (e.g., the pictures in a) to another (e.g., the toys in b).

Horst (2013) analyzed methodological aspects in research on storybook reading with the focus on word learning and considered whether it is best to use commercially available or purpose-written books. She recommends using purpose-written storybooks, because they allow researchers to control for unintended differences between conditions. Therefore, we used specially designed picture books in our training procedure (**Figure 2**, top). The training items were put together in a colorful folder to form an eight-page picture book (four for the *behind* and four for the *next to* relation). The items were presented in randomized order (see Supplementary Material for a complete list).

#### Pre- and Posttest Items

The test materials took the form of a picture-selection task and consisted of 12 standard-sized (A4) sheets of paper, each depicting four photographs of objects with names already common in children’s lexicons. Each photograph showed the same three single objects (a trajector object [tr], a landmark object [lm], and a distractor [dis] object, see **Figure 1**) arranged into four different spatial relationships: the particular relationship being trained plus three other relationships. The positions of the target relations were randomized. For example, a bench was chosen because the relations *on* and *under* are also plausible with it. One-half of the items included animate objects and the other half included inanimate objects to create a high diversity of stimuli. **Figure 1** depicts two different categories of items created to assess the children’s ability to retain or generalize the new words: trained and untrained. Whereas trained items were applied in each testing phase as well as in the training phase (e.g., a rabbit, a cat, and a hutch) and assessed children’s retention ability, the untrained items appeared first at posttests and were presented to measure children’s ability to extend the spatial relations they had learned. The children were not exposed to them before the test. Untrained items were subdivided into two further test sets (see **Figure 1**) to account for children’s canonical knowledge of landmark objects (Rohlfing, 2006): one group of untrained test items contained objects that can be related to each other (e.g., a boy as well as a bird can sit in a boat), because it contained a trajector object (e.g., a boy), a landmark object (e.g., a boat), and a distractor object (e.g., a bird). Another group of items contained objects that do not afford particular spatial relationships because they all are trajector objects (e.g., an apple, a pear, and a strawberry).

#### Picture-Selection Task

In this forced-choice comprehension task, the experimenter placed a book with the test items (the number of items depended on the test phase, see below) in front of the child on the table. Each item consisted of four photographs of objects showing four different spatial relations (e.g., *on, behind, next to*, and *in front of*). After naming the depicted objects, the experimenter instructed the child to indicate the target picture. Each page included a total of three instructions including one for each target preposition (*behind* and *next to*) as well as one for the non-trained spatial relation *on* serving as a control variable. The instructions were as in the following example:

Ich habe dir ein paar Bilder mitgebracht. Wir machen das jetzt so: Ich erzähle dir etwas und du zeigst mir das richtige Bild dazu. Es ist immer nur ein Bild richtig. Wir probieren das einmal aus. Hier sind ein Hase, eine Katze und ein Stall. Jetzt zeig mir bitte mal das Bild: der Hase steht auf/hinter/neben dem Stall.
[I brought some pictures for you. We are now going to do the following: I’ll tell you something and you show me which is the right picture. There is always only one right picture. Let’s try it out once. Here is a rabbit, a cat, and a hutch. Now please show me the picture in which the rabbit is *on/behind/next to* the hutch.]

The child’s task was then to indicate the correct picture. The child’s response was acknowledged without any comment on the result.

#### Acting-Out Task

The acting-out task assessed children’s task transfer (see below) and was an open-ended task: the children were asked to arrange the relations with toy objects in line with the following instruction:

Ich habe dir Spielzeug mitgebracht. Wir machen das jetzt so: Ich hole die Sachen der Reihe nach auf den Tisch und wir schauen sie uns gemeinsam an. Hier sind ein Hase, eine Katze und ein Stall. Und jetzt stell du mal den Hasen auf/hinter/neben den Stall. Prima!
[I brought some toys for you. We are now going to do the following: I’ll put some things on the table one after the other and we’ll have a look at them together. Here is a rabbit, a cat, and a hutch. Now please, put the rabbit *on/behind/next to* the hutch. Great!]

In addition to carrying out the testing procedure, we collected some further relevant variables. First, during the second visit, we tested children’s individual language skills with the *SETK-2* (*Sprachentwicklungstest für zweijährige* Kinder, Grimm, 2000). We used the results of the sentence comprehension subtest because it was most germane to our interest in the child’s ability to benefit from narrative structure. Second, we asked parents to complete questionnaires tapping demographics, children’s use of spatial language (see Supplementary Material), and picture book reading experience. These included a *language survey* (Grimminger et al., 2010) with which parents rated their child’s receptive and productive skills for spatial relations. This allowed us to determine whether children already comprehended or produced the target prepositions *behind* and *next to* upon enrollment (see Supplementary Material).

### Procedure

The training study followed a pretest–posttest design with two posttests to access slow mapping, that is, robust word learning in children (Carey, 1978; Horst and Samuelson, 2008). Participants visited the lab a total of four times (see **Table 1** for an overview and the duration of sessions). All visits were scheduled within 2 weeks with the proviso of only one visit per participant per day and a maximum of 14 days between the first and last visit. As long as participants met this proviso, the exact duration and span between test dates was not measured for each participant. Thus, the time range for all participants was a minimum of 4 and a maximum of 14 days (in most cases, 2 days within 1 week) depending on parents’ availability. Until the last visit, parents remained unaware of the experimental assumptions and were asked not to practice any of the tasks they saw with their child.

**Table 1:** Overview of the items applied in each testing and training phase including the amount of instructions.

Visit	Testing phase	Items	Instructions
1 (50 min)	PRETEST Picture-selection task	Rabbit/hutch Spoon/cup	Six instructions in total: Three instructions for each item: *on, behind*, and *next to*
	Acting-out task	Rabbit/hutch Spoon/cup	Six instructions in total: Three instructions for each item: *on, behind*, and *next to*
	TRAINING Training 1	Rabbit/hutch *behind* Rabbit/hutch *next to* Spoon/cup *behind* Spoon/cup *next to* Girl/bench *behind* Girl/bench *next to* Umbrella/wardrobe *behind* Umbrella/wardrobe *next to*	Presentation in randomized order
	IMMEDIATE POSTTEST Picture-selection task	Rabbit/hutch (trained) Spoon/cup (trained) Four untrained set of pictures (randomized choice)	18 instructions in total: Three instructions for each item: *on, behind*, and *next to*
	Acting-out task	Rabbit/hutch (trained) Two untrained sets of toys (randomly chosen from the untrained items applied in the picture-selection task)	Nine instructions in total: Three instructions for each item: *on, behind*, and *next to*

2 (40 min) + 3 (15 min)	TRAINING Training 2 and 3	Repetition of training 1	Presentation in randomized order

4 (30 min)	DELAYED POSTTEST Picture-selection task	Rabbit/hutch (trained) Spoon/cup (trained) Girl/bench (trained) Umbrella/wardrobe (trained) Four untrained set of pictures (randomized choice)	24 instructions in total: Three instructions for each item: *on, behind*, and *next to*
	Acting-out task	Rabbit/hutch (trained) Two untrained set of toys (randomly chosen from the untrained items applied in the picture-selection task)	Nine instructions in total: Three instructions for each item: *on, behind*, and *next to*

*The delay was a minimum of 1 day between visits 1 and 2 and a maximum of 14 days between visits 1 and 4.*

The dependent variable was children’s comprehension of the target spatial prepositions operationalized by their performance in a forced-choice picture-selection task. The study followed a 3 × 2 design with *time* (pretest, immediate posttest, and delayed posttest) as within-subject variable and *group* (narrative input structure and unconnected speech) as between-subject variable. To investigate the effects of children’s overall sentence understanding on their learning performance, their comprehension of sentences was assessed with a subtest of the SETK-2 (Grimm, 2000).

The study took place in a Dialoglab room at CITEC, Bielefeld University (Nachtigäller, 2016). For the study, no ethics approval was obtained, because at the time the experiments were conducted, the university did not require ethics approval. However, the study followed the ethical standards established in the Declaration of Helsinki (1964) and its successive emendations: all participants were paid volunteers. Parents of the subjects were informed about the aims, experimental procedures and possible risks of the study, and a written freely given informed consent was required from all of the parents prior to the study. Parents were present in experiments with their children and were free to withdraw their consent to participation at any time and for whatever reason. To ensure confidentiality, the data analysis was done on the language group as a whole.

For the children, a warm-up period was followed by both the testing and the training procedures carried out with the experimenter and child sitting at 90° to each other on two sides of a table. A box with all stimuli was placed next to the experimenter out of reach of the child so that each item could be taken out separately and placed on the table in randomized order. Although parents were allowed to be present in the room, they were not involved in the testing and training procedures and they were instructed not to interact with their child during these procedures. Four different experimenters conducted the study in both groups. Experimenters received advance training on how to instruct and complete the testing phases and how exactly to train children in order to reach an optimal level of standardization.

#### Pretest

The pretest provided a necessary baseline of children’s performance *on* the spatial relations *behind* and *next to*. In addition, the spatial relation *on* served as a control and distractor. This was not trained. We tested children’s performance with two sets of pictures and two sets of toys.

#### Training

The training procedure was designed as a picture book reading scenario. The experimenter opened this phase by instructing the child as follows: “Now, we are going to do something else: I’ll show you lots of interesting pictures and I’ll tell you stories about them. And you, you listen very carefully, okay?” A book including all training pictures in randomized order was placed on the table (see Supplementary Material for complete lists of the verbal input provided for each spatial preposition). To enhance engagement, the child was allowed to turn the pages. Groups differed only in terms of the verbal input to which the children were exposed (narrative structure vs. unconnected speech). After listening to each passage, the child was free to comment briefly on the items, but was not asked to do so. Also, the experimenter tried to reduce talking about items to a minimum during this phase. Following findings in word learning studies suggesting that distributed exposure to new words over time is more effective for learning than massed exposure at a single time point (Tomasello, 2003; McGregor et al., 2007; Horst, 2013), we carried out three training sessions on different days in which children were provided with the same stories in randomized order.

Short passages were created for all groups; each containing four sentences and between 31 and 36 words. The number of words per passage did not differ between conditions (*U* = 19.500, *p* = 0.195). In both conditions, each target preposition was named once per page/training item and on four different pages/training items per session. This resulted in a total of 12 namings over all three training sessions (see Supplementary Material).

In the *narrative group*, passages were coherent sentences with a temporal and causal structure. Whereas the first sentence described a general situation introduction (e.g., about the weather), the second and third sentences introduced the trajector and landmark objects involved in the narrative and depicted a motivation for an action. Finally, in the fourth and last sentence, the action was carried out resulting in a consequence. The target preposition was introduced and named once in this last sentence. Corresponding pictures collected in a picture book depicted the end state of the short narratives. Compared to our previous study (Nachtigäller et al., 2013), the narratives were longer at four sentences. Though lacking all characteristics of a complete narrative, this operationalization aimed to model real narrative structures that were age-appropriate for 27- to 29-month-old children.

In the *unconnected speech group*, the passages contained deictic expressions (cf. Hayne and Herbert, 2004) and some adjectives describing the items; but there were no temporal or causal relationships. The adjectives were added to enhance the ecological validity of the control input and to prevent children in this condition from getting bored with the input. Most importantly, trajector and landmark objects were also introduced in the second and third sentences and target prepositions were named in the last sentence. Thus, the target prepositions were named equally often and positioned at the same place in the input as in the experimental condition.

In the *object group*, the children received the same narrative input as in the narrative group but were trained with toy objects rather than pictures. For their training, the experimenter placed the objects corresponding to each set on the table in an already prepared static arrangement that paralleled the arrangement in the pictures in the picture group (see **Figure 1**) and then told the story. Thus, instead of seeing, for example, the bench being put on the table first and then the girl arranged close to it, the child saw the experimenter putting the bench and the girl already in a fixed relation to each other on the table. This way, a movement of the trajector object was avoided that could potentially highlight the target relation and thus differ from a stimulus presentation in the picture group. While the story was being told, the experimenter pointed to each object and named it. Items were finally placed at the center of the table where the child could see but could not reach them. Apart from the training, input was reduced to a minimum. After each narrative, the child was allowed to touch the items or play with them shortly (we evaluate this decision in the Section “Discussion”). Sets were presented in randomized order at each training session.

#### Immediate Posttest

We tested children’s retention performance by asking them to select the appropriate relation when presented with items familiar from pretest and training. To test for generalization, children were asked to select the appropriate picture matching the requested spatial relation from four pictures on which untrained items were presented. Another crucial test for generalization assessed children’s ability to transfer their word knowledge to another context. For this purpose, children in both groups were asked to act out the requested relation with toys. The presentation of items and the order of instructing the spatial prepositions *on, behind*, and *next to* was randomized. In the following, we will focus only on the trained item set. The idea behind this procedure was that testing with the material familiar from training should help the children to recognize and recall their word knowledge. This is because children learn best when retrieval and encoding conditions are similar (Goldenberg and Sandhofer, 2013). In contrast, the untrained task with other material but similar objects was thought to impose different but feasible demands on children’s comprehension of the spatial prepositions. This created a task transfer (see **Figure 2**) to test the children’s slow mapping ability: although the items were familiar from training, children had to recognize them with different materials and perform within tasks imposing different requirements.

#### Delayed Posttest

Children’s retention performance was tested with four sets including trained items that were familiar from the pretest, training, and immediate posttest. Again, children’s ability to generalize word knowledge was tested with four untrained items. In addition, the ability to transfer the word knowledge to an untrained task (from pointing to pictures to acting out on objects) was assessed with three items sets.

**Table 1** gives an overview of the items applied in each testing and training phase.

### Coding

In the picture-selection task, the child had a 25% chance of guessing correctly. If the child pointed to the correct picture (i.e., depicting the instructed spatial preposition), she or he was awarded one point. Zero points were given if the child pointed to one of the other three pictures. The acting-out task was assigned zero points when a child performed the wrong relation or did not react at all; one point when a child answered with the right relation that was achieved by an atypical approach (e.g., using the wrong trajector or landmark); and two points for a correct performance. This scale reflects a development of semantic knowledge acknowledging children’s intermediate understanding of prepositions in the acting-out task. In other words, some children acted according to an incomplete knowledge about which action is required as a response to the instruction. Mean values were calculated on children’s performance with the relations *behind* and *next to* together for each testing time (pretest, immediate test, and delayed posttest), item category (trained/untrained), and task (picture selection and acting-out). Results are presented in percentages of correct answers.

A second rater coded four participants who had been selected randomly from each condition (corresponding to approximately 25% of the data). Cohen’s kappa of 0.91 indicated a high inter-rater agreement for the whole testing procedure. In detail, Cohen’s kappa was 0.89 for the pretest, 0.90 for the immediate posttest, and 0.92 for the delayed posttest.

## Results

In the following, we compare children’s performance in the narrative group to the unconnected speech group. The analyses for the object group will be described separately, because different scales underlie the dependent variables: thus, the results cannot be compared directly. All data are presented in **Table 2**.

**Table 2:** Mean percentage (and standard errors in parentheses) of children’s correct retention performance in all groups and tests; chance level of correct performance is 25%.

	Groups		
	Narratives with pictures	Unconnected speech with pictures	Narratives with objects
**TESTS**			
RETENTION: Testing understanding with items familiar from training			
Pretest	32.81 (5.82)	38.33 (6.01)	10.83 (4.20)
Immediate test	40.63 (7.79)	33.33 (8.05)	19.17 (7.80)
Delayed test	50.00 (5.76)	35.00 (5.94)	23.75 (5.66)
GENERALIZATION: Testing understanding with untrained items			
Pretest	35.00 (5.97)	37.50 (6.18)	10.83 (4.02)
Immediate test	37.50 (5.11)	26.79 (5.29)	16.25 (3.60)
Delayed test	35.00 (6.03)	35.71 (6.24)	23.33 (5.93)
TRANSFER: Testing understanding with untrained material but items familiar from training			
Pretest	30.36 (9.39)	23.33 (9.07)	26.67 (7.10)
Immediate test	35.71 (10.63)	23.33 (10.27)	23.33 (6.67)
Delayed test	35.71 (10.48)	23.33 (10.12)	33.33 (9.34)
TRANSFER AND GENERALIZATION: Testing understanding with untrained items and untrained material			
Pretest	28.33 (9.11)	25.00 (9.43)	26.67 (7.10)
Immediate test	39.17 (7.68)	20.54 (7.95)	16.67 (6.30)
Delayed test	45.00 (7.22)	19.64 (7.22)	23.33 (5.93)

*Note that scores in the object groups are based on a dependent variable different from the dependent variable in the other groups.*

### Learning with Trained Item

**FIGURE 3:**
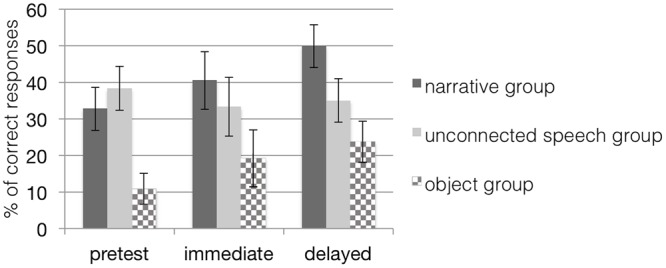
**Mean percentage (and standard error bars) of children’s correct *retention* performance in all groups when tested with trained items; chance level of correct performance is 25%.** Note that scores in the object groups are based on a dependent variable different from the dependent variable in the other groups. The narrative group received training with narratives on picture material; the unconnected speech group was trained on pictures but without narratives; the object group was trained with narratives on objects.

First, we investigated the learning effect over the course of the study in the two groups learning with pictures. According to a 3 (time) × 2 (group) repeated measures ANOVA, there were no main effects of time, *F*(2,28) = 1.92, *p* = 0.16, or group, *F*(1,29) = 0.70, *p* = 0.41, but a significant interaction effect of time and group, *F*(2,28) = 3.26, *p* = 0.05, η^2^ = 0.19, with a medium effect size (see **Figure 3**). To analyze the interaction effect in more detail, a subsequent test of simple effects revealed a significant main effect of time, *F*(2,14) = 4.33, *p* = 0.03, due to a significant difference between pretest and delayed posttest (*p* = 0.03) for children in the narrative group. There were no significant differences between testing times for children in the unconnected speech group. Children in both groups started with a comparable performance level at pretest, but children from the narrative group improved their performance over pretest and strengthened their comprehension of the target words at both posttests. In contrast, the learning curve of the control group remained on the baseline level. Thus, children in the unconnected speech group did not seem to benefit from their input. Instead, a closer look at the mean performance of children in this control group even revealed a slight decrease from pretest to immediate posttest. Results thus confirmed our hypothesis: children in the narrative training group learned and retained the target words better over the course of several days than children in the control group with unconnected speech when both were tested with sets containing trained items in the picture-selection task.

Our analyses of children’s retention performance in the object group when performing the acting-out task (see **Table 2**) revealed no significant main effect of time, *F*(2,13) = 1.51, *p* = 0.26, η^2^ = 0.19, indicating that children trained with objects did not improve in understanding the trained spatial prepositions.

In addition to our analyses, we also considered children’s errors in their answering behavior to reveal whether learners in one group made errors of a particular category more frequently than children in other groups. For this purpose, in cases in which a child did not provide a correct answer in the picture-selection task, we classified the answering behavior according to three categories: (a) no picture was selected (no relation), (b) another relation was selected (other relation), (c) the other trained, but not instructed relation (e.g., *next to* instead of *behind*) was selected (trained relation). We found that children in the narrative group barely made errors of the category “(a) no picture selected,” indicating that they always answered by selecting a picture, whereas errors in all three categories could be found in the answering behavior of children from the unconnected speech group.

For the object group, in which children were instructed to act out a relation, errors were categorized into the same three categories. The error analysis revealed that children in the object group hardly ever acted out the other trained relation, and did so even less in cases of the *behind* relation. We mostly observed errors of category “(b) another relation.” Note, however, that this category included a large number of possible relations.

In addition to the descriptive analysis, we tested the hypothesis that children might have confounded the requested relation with the other learned target relation. For this purpose, children’s answers in the picture-selection task were subjected to a 3 (time) × 2 (group: narrative and unconnected speech) × 2 (trained relation: requested vs. not-requested) repeated measures ANOVA. This enabled us to differentiate between whether children’s choices were due to the instruction or to the experienced training (in the case a child performed *next to* when *behind* was instructed).

There was a strong main effect of trained relation, *F*(1,29) = 20.03, *p* < 0.001, η^2^ = 0.41, according to which children responded with the requested relation (38.35 %) more often than with the other trained relation (19.1%). The analysis of children’s performance in the object group when performing the acting-out task revealed only a statistical trend toward a main effect of relation, *F*(1,14) = 3.6, *p* = 0.08, η^2^ = 0.20. This difference is likely to be due to smaller numbers of performances in the object group.

In sum, the additional error analysis did not support the hypothesis that learning one preposition might have influenced learning the other trained preposition. By revealing that children did not confound the requested with the other trained preposition, we provided further support for the finding that children in the narrative group retained the target words better over the course of the study compared to the unconnected speech or object group.

### Generalization to Untrained Items

Generalization to untrained items is assumed to be more difficult than performance on trained sets, because the context provides fewer scaffolds. Consequently, a robust semantic representation is needed to support the child’s accomplishment of this task. We assumed that children who were trained via narratives would be better able to generalize their newly acquired preposition to sets with untrained items than children in the control condition with unconnected speech.

**FIGURE 4:**
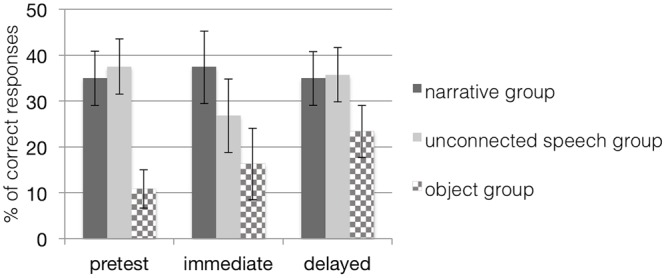
**Mean percentage (and standard error bars) of children’s *generalization* performance in all conditions when tested with untrained items; chance level of correct performance is 25%.** Note that scores in the object groups are based on a dependent variable different from the dependent variable in the other groups. The narrative group received training with narratives on picture material; the unconnected speech group was trained on pictures but without narratives; the object group was trained with narratives on objects.

A 3 (time) × 2 (group) repeated measures ANOVA revealed neither a main effect of time, *F*(2,26) = 0.35, *p* = 0.71, nor a main effect of group, *F*(1,27) = 0.20, *p* = 0.66, nor an interaction effect of time and group, *F*(2,26) = 0.94, *p* = 0.40 (see **Table 2**) indicating that children were unable to generalize their acquired word knowledge to untrained items at posttests in either the narrative or the unconnected speech condition (see **Figure 4**). Thus, results did not confirm our hypothesis that children at the age of 28 months would perform better by more effectively connecting cues from the semantic context provided in the narrative group to their established semantic network. This result was echoed for the object group as the analyses revealed no significant main effect of time, *F*(2,13) = 1.85, *p* = 0.20, η^2^ = 0.22, indicating that the narrative training presented with real objects did not help the children to generalize their acquired word knowledge to untrained items at posttests.

### Generalization to an Untrained Task (Transfer-Task)

A crucial extension of common generalization tests is our investigation of whether word meaning acquired in the context of picture book reading could be applied successfully in another situation and whether the narrative condition supports this task transfer in learning. Even if the objects deployed were familiar to children (because they had already seen them on pictures), the task imposed untrained pragmatic and cognitive demands on them.

**FIGURE 5:**
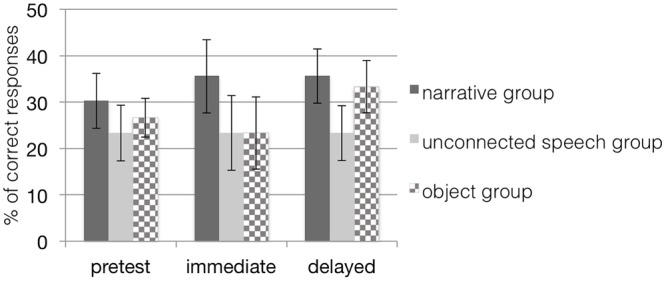
**Mean percentage (and standard error bars) of children’s *generalization* performance to an untrained task (*task transfer*) in both conditions when tested with trained items.** Note that scores in the object groups are based on a dependent variable different from the dependent variable in the other groups. The narrative group received training with narratives on picture material; the unconnected speech group was trained on pictures but without narratives; the object group was trained with narratives on objects.

A 3 (time) × 2 (group) repeated measures ANOVA revealed neither a main effect of time, *F*(2,26) = 0.16, *p* = 0.86, η^2^ = 0.01, nor a main effect of group, *F*(1,27) = 0.73, *p* = 0.40, nor an interaction effect of time and group, *F*(2,26) = 0.16, *p* = 0.86, η^2^ = 0.01, indicating that children in neither the narrative nor the unconnected speech condition were able to generalize their acquired word knowledge to trained items in a untrained task at posttests (see **Figure 5**). Thus, results did not confirm our hypothesis that children at the age of 28 months would perform better by more effectively connecting cues from the semantic context provided in the narrative group to their established semantic network as tested in an untrained pragmatic context.

The analyses from the object group also revealed no significant main effect of time, *F*(2,13) = 0.51, *p* = 0.61. Accordingly, the narrative training with objects did not influence children’s performance in the picture-selection task with trained items when compared to their baseline performance at pretest.

Taking the results together, our hypothesis that narrative structure in the input would strengthen the semantic memories to such an extent that they could be retrieved for a task transfer was not confirmed: in the posttest, children in both narrative groups (trained with pictures and objects) showed a comparable performance to the pretest and did not improve their performance in transferring their word knowledge to untrained materials.

### Generalization to Untrained Items in an Untrained Task

The most difficult test for children’s generalization performance was the transfer to untrained items presented in an untrained task, because children were expected to be minimally scaffolded from the context, so that the stimuli as well as the task afforded a transfer of knowledge.

**FIGURE 6:**
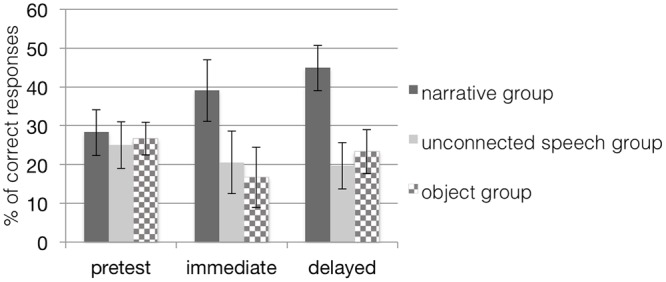
**Mean percentage (and standard error bars) of children’s *generalization* performance to untrained items (*task transfer*) when tested in an untrained task in both conditions.** Note that scores in the object groups are based on a dependent variable different from the dependent variable in the other groups. The narrative group received training with narratives on picture material; the unconnected speech group was trained on pictures but without narratives; the object group was trained with narratives on objects.

Again, we conducted a 3 (time) × 2 (group) repeated measures ANOVA revealing neither a main effect of time, *F*(2,26) = 0.73, *p* = 0.49, nor a main effect of group, *F*(1,27) = 2.42, *p* = 0.13, but a marginal interaction effect of time and group, *F*(2,26) = 2.85, *p* = 0.08, η^2^ = 0.18, with a medium effect size (see **Figure 6**). To analyze the interaction effect in more detail, a subsequent test of simple effects revealed a marginal main effect of time, *F*(2,13) = 3.49, *p* = 0.06, due to a significant difference between pretest and delayed posttest (*p* = 0.02) for children in the narrative condition. There were no significant differences between testing times for children in the unconnected speech condition. Our results thus indicate a small advantage for children in the narrative group when presented with unfamiliar items in an unfamiliar task.

For the object group, when testing children’s performance with untrained items in an untrained task (picture selection), results revealed no significant main effect of time, *F*(2,13) = 0.80, *p* = 0.47, indicating that children in the object group could not benefit from the training when tested for their ability to generalize their word knowledge to untrained items in an untrained task (see **Table 2**). This result contrasts with the statistical trend notable in the narrative group trained with pictures.

### Relationship of Word Learning and Language Skills

In a recent study (Nachtigäller et al., 2013), we found that 2-year-old children with advanced expressive language skills were in a good position to benefit from narrative context in order to generalize a learned meaning to untrained items. Thus, in our further analysis, we tested whether sentence comprehension in general would correlate positively with the amount of new word learning. In the narrative group, children’s performance immediately after training was not related to their sentence comprehension: *r* = 0.46, *N* = 15, *p* = 0.08 (for retention performance) and *r* = 0.44, *N* = 15, *p* = 0.10 (for generalization). However, at the delayed posttest, their sentence comprehension scores showed a significant positive correlation with retention performance for trained items (*r* = 0.85, *N* = 15, *p* < 0.01), with generalization performance to untrained items (*r* = 0.68, *N* = 14, *p* < 0.01) and with generalization to untrained material; that is toys (*r* = 0.71, *N* = 15, *p* < 0.01) that were already known from the pictures. These findings indicate that the higher the children’s level of sentence comprehension, the more they are able to benefit from the narrative training.

In the control group with unconnected speech, although the sentence comprehension scores of the children also correlated significantly with retention performance at the delayed posttest (*r* = 0.66, *N* = 15, *p* < 0.01) but not at immediate posttest (*r* = 0.39, *N* = 15, *p* = 0.15), we found no significant relation between children’s sentence comprehension scores and scores on generalization to untrained items either at the immediate posttest (*r* = 0.19, *N* = 15, *p* = 0.50) or at the delayed posttest (*r* = 0.25, *N* = 14, *p* = 0.38). Hence, those children with advanced sentence comprehension in the control group retained words better at posttests, but their language skills seemed to be unrelated to the ability to generalize the acquired word knowledge to untrained items.

For children in the object group, sentence comprehension at the immediate posttest did not correlate with performance on either the acting-out task (*r* = 0.13, *N* = 15, *p* = 0.65) or the task transfer with untrained materials (*r* = 0.04, *N* = 15, *p* = 0.88). The same was true for the delayed posttest: there was no significant correlation with either acting-out (*r* = 0.38, *N* = 15, *p* = 0.17) or task transfer with untrained materials (*r* = 0.39, *N* = 15, *p* = 0.15).

## Discussion

Our study was designed to reveal (a) the support of narratives in organizing semantic networks for learning spatial prepositions and (b) whether spatial prepositions can be learned within the context of picture book readings. Furthermore, we provided an extended investigation of the slow mapping process analyzing (c) how far the acquired word meaning can be generalized to a context different from training conditions.

### The Benefit of Narratives

We investigated whether a narrative input structure—in comparison to input with no semantic structure—presented within a picture book reading activity can provide 28-month-old children with a beneficial context for learning the spatial prepositions *behind* and *next to*. We were motivated by studies suggesting that learners of this age will show a bias toward semantically related words because they are more experienced in learning language and should have established a system of meanings (Gershkoff-Stowe, 2002; Arias-Trejo and Plunkett, 2009) enabling a direct connection of the novel meaning to the established relationships among different words. We expected the general support provided by the greater semantic network to be reflected in children’s ability to retain and generalize the novel words in the long-term.

Testing children’s performance for *retention*, we found that although the control and the narrative group started from a comparable level at pretests, children in the narrative group improved their performance on the immediate and the delayed posttest, whereas children in the control group, trained with unconnected speech, remained on the same performance level as in the pretest. This finding was supported by our error analysis indicating that children did not confound the requested preposition with the other trained one, which is in line with Munro et al.’s (2012) study that found no support for errors of semantic category either.

The narrative training turned out to be beneficial for children’s long-term retention of gains in word knowledge. Our finding accords with other research demonstrating the effects on children’s later memory retrieval of specific types of “semantic enrichment” (Capone Singleton, 2012, p. 279) such as causal descriptions of new labels (Booth, 2009), narration during encoding (Hayne and Herbert, 2004; Nachtigäller et al., 2013), and topic coherence (Horowitz and Frank, 2015).

In contrast to our previous findings with younger children (Nachtigäller et al., 2013), in the current study, the benefit of narrative structure on word learning was not statistically reliable until the delayed posttest captured the slow mapping process; the pretest and immediate posttests did not differ significantly. This finding is in line with other research reporting that an enhancement of newly learned words became prominent after days or weeks rather than immediately after training (Booth, 2009; McGregor et al., 2009). This effect is probably due to multiple repetitions of our stories over three training sessions on three different days. According to Horst (2013), repeated exposure to storybook texts and illustrations leads to a robust representation of a new word, because “such contextual repetition helps lower the attentional demands of word learning” (p. 2). Consolidation is another possibility. Wojcik (2013) points out that after encoding a new word, a perceptual trace is translated into a cortical memory trace that can be maintained over a longer period of time. This process is called consolidation and it occurs with the passage of time without any need for additional direct training exposure (Munro et al., 2012). Landau (1994) claims that a representation of a spatial relation is more schematic than a representation of an object and might thus need more consolidation time. In the current slow mapping study, there was always a minimum of 1 day, in most cases several days, between the last training session and the delayed posttest. The children’s memory for the newly learned spatial preposition might have (a) improved in this period of recovery from training—an effect also known from other language learning studies (Booth, 2009; Rost, 2011); or (b) needed time to consolidate with the understanding of narratives. Further research might investigate the minimal time required for consolidation processes by varying the duration until a learning effect can be observed.

Whereas we found that, regardless of their language skills, children can benefit from the semantic structure in the input when testing whether they *retain* gains in word knowledge, we were unable to find a clear causal effect of narrative input on children’s ability to *generalize* their word knowledge to unfamiliar items. Here, we found that in contrast to retention, the ability to generalize as a robust form of learning correlated with children’s language skills. Even though our design applied a multiday procedure to access children’s generalization performance, we think that the word learning process goes beyond this scheduled period. It is possible that we failed to find generalization effects in the narrative group because we assessed children just when they were in the middle of “weaving” the novel meaning of the spatial prepositions into their semantic network. Whereas simulation processes might be responsible for retention abilities to emerge, generalization in children could require deeper consolidation processes initiated by cross-situational learning to abstract episodic memories from their original context and to enable the discernment of similarities across situations. Our correlational findings support this speculation, because children who were more advanced in their language skills proceeded faster through this learning period, especially when scaffolded by the semantic context in the narrative input. Also Nachtigäller et al. (2013) found that the children who generalized to unfamiliar items were those whose productive vocabulary was reported to be more advanced than their studied peers. What makes these children learn faster is an open question. One possibility is that greater experience with language in general allows children with a stronger vocabulary to better fill the units of the events with content and thus to retrieve a richer representation or to induce a more comprehensive mental simulation (Edwards et al., 2004; Gershkoff-Stowe and Hahn, 2007). This interpretation would be in line with embodied theories and should be reflected by a decline in individual differences in learning with growing language experience. Another, not exclusive, possibility is that children with growing language experience have developed cognitive abilities to faster assimilate new words. Here one can speculate that the cognitive strength of these children lies in the ability (a) to cope with fewer examples of a referential event (i.e., to know more intuitively which relevant aspects of a situation to memorize when being taught a word of a particular class; Thom and Sandhofer, 2009), (b) to be faster in integrating structured aspects of an ongoing situation with already existing semantic networks (i.e., being able to induce mental simulation on the basis of word memories), and (c) to be better at consolidating particular aspects from repeated exposure to the exemplars (i.e., to utilize knowledge achieved via retention) to form robust semantic memories.

Semantic theories, thus, have to incorporate the developmental fact that language knowledge is accumulating and it is likely that this leads to changes in cognitive operations (Bjorklund, 1987). In this sense, for example, an event can be simulated from the narrative input only when children understand the meaning of the full sentence. For children with sparse sentence understanding, these mechanisms do not yet seem to be functioning.

### The Benefit of Pictures

Can spatial prepositions be learned from pictures? We evaluated this question by training a control group of children with narratives while showing them toy objects rather than pictures. Note that the two groups could not be compared directly due to a different dependent variable: a picture-selection task in the picture group and an acting-out task in the object group.

Surprisingly and contrary to the assumption that a concrete experience of the spatial relationship is required in order to learn a spatial preposition, children in the object group did not learn from the training although they listened equally often to the new prepositions embedded within a semantically structured input. This difference may well reflect the different demands in the tasks of the test: The acting-out task with real objects is complex, because it requires a child to choose two objects from a selection of three and to arrange them in a requested manner. In addition, in order to perform correctly, learners had to inhibit the present objects’ affordances (e.g., the canonical action of putting the rabbit *into* the hutch as afforded by its concavity). This problem was notified by DeLoache (1991) for younger children. In comparing how children discern the symbolic nature of pictures with models, she observed that children gradually (not until 3 years of age) become capable of inhibiting the object’s known function in order to respond to an object in a novel way. In contrast, by looking at the possible relations presented in the picture-selection task, the trained relation received more cues that aid recognition (Capone and McGregor, 2005; McGregor et al., 2009). In this way, a weaker semantic representation might have been sufficient to solve this task.

Another factor contributing to the missing learning effect in the object group might have been the design of the training: in our previous study, younger children learned to understand the spatial preposition *under* with real toys (Nachtigäller et al., 2013) but were trained with a *dynamic* demonstration of the spatial configuration that was labeled synchronously with the spatial preposition. In the present study, in contrast, the objects were arranged *statically* during training to parallel the static arrangement depicted in the pictures. We allowed the children to play with the objects only after they had listened to the stories to ensure that the object group would not gain an advantage from getting more involved in the objects through manipulation than the picture group. However, our decision might have obliged the children from the object group to inhibit their impulse to grasp and manipulate the objects for the whole duration of the narrative input. Thus, it is possible—and the analysis of errors in children’s answers supports this point—that the presentation distracted from the input and competed with the children’s canonical manipulative object knowledge (Rohlfing, 2006).

Clearly, all the aforementioned factors might have increased the difficulty of the acting-out task and contributed to the lack of retention performance in this condition. From our results, we can thus draw the limited conclusion that static configurations with real objects seem to provide a child with a less appropriate learning context that does not lead to long-term retention compared to pictures supporting the content of a narrative. In this sense, when it comes to slow mapping, pictures seem to be an effective means of supporting the content of a narrative, because the test might be easier to perform and the manipulation of objects is not afforded in this situation. This finding amplifies other positive effects reported on word retention: Repetition of this situation is easily possible (Horst et al., 2011; Nachtigäller and Rohlfing, 2011b) and children become tuned to a particular kind of conversation (involving more generic and ostensive labeling phrases) that fosters the categorization of objects (Gelman et al., 2005). The fact that children trained with pictures showed a significant learning of the newly acquired spatial prepositions only at the delayed posttest indicates that word knowledge was consolidated in the time between training and testing—an effect also reported in Booth (2009).

### Transfer of Learning from Pictures

An interesting and innovative aspect of our study was the attempt to further investigate the depth of the word knowledge acquired in the context of book reading. We attempted this by testing the children’s ability to transfer their understanding to an untrained task. In contrast to previous studies (Ganea et al., 2008; Ganea and Canfield, 2015) in which transfer ability was attested to 15-month-olds for nouns, our results on spatial prepositions do not speak to children’s transfer ability in either the narrative or the object group. This null result is remarkable given that we designed the task transfer to be actually feasible for the learners: the pictures were faithful to the objects, and Ganea et al. (2008) report how iconicity scaffolds children’s transfer efforts. Whereas the picture group showed a trend toward a task transfer when tested with objects that were unfamiliar, this effect was not noticeable in the object group. This suggests, again, that for the understanding of spatial prepositions—in contrast to nouns, in which case children can demonstrate their understanding of real objects and pictures uniformly by pointing to the referents—different tasks go hand in hand with imposing distinct cognitive demands on the children: different materials are likely to evoke different perceptions in children, that is, when faced with objects, they will manipulate them (DeLoache, 1991), and they are obviously distracted when this possibility is not available. Children eventually behave differently with pictures, though they manipulate the book as an object a lot at younger ages by, for example, chewing on it, folding it, and so forth. DeLoache (1991, p. 750) points to the possibility that children at the age studied here respond to the pictures in a different way than adults who know that pictures have an “informative relation to reality.” This different perception of pictures might advance children in learning from this material.

Recall that when it comes to children’s language skills as operationalized by their sentence understanding, our analyses revealed a correlation with the word learning performance in the picture group. Yet, we found a lack of correlations in the object condition, which, again, might be a reflection of the task difficulty being greater in the acting-out task than the picture-selection task and an indication that other factors (such as affordances of objects) are distracting the children’s learning processes.

Taking the findings from the narrative picture and object group together, our results are in line with Madole and Oakes (1999) who state that children’s categorical performance during an experiment depends on the task demands and on the stimuli used. A given performance does not necessarily reflect a stable representation; it may rather be a reflection of the child’s ability to handle the task demands. The authors suggest that similar fundamental cognitive processes may well come into operation when dealing with different tasks addressing the same topic, but that the tasks nonetheless imply different cognitive demands (Madole and Oakes, 1999). In the present study, a pretest served as a baseline measure in order to control for the different task demands. It seems that very specific training (i.e., with particular items and within a particular task) supports children’s slow mapping but interfaces with the children’s experience in processing linguistic and task demands. In this sense, the development of the semantic network does not seem to be an exclusive product of overall exposure to language. Instead, the initial representation also binds memories about the pragmatic circumstances in which the word occurs (Binder and Desai, 2011; Rohlfing et al., 2016). This can function as a cue when retrieving the word knowledge. Word knowledge about spatial prepositions contains information about spatial relations. Concluding from the results of her developmental research, Gentner (1988) suggests that cognitive abilities that are relevant for discerning relations among objects can first be observed in familiar domains. The context of pictures might be such a familiar domain in which children are more likely to apply particular cognitive operations. As suggested already above, Gelman et al. (2005) found that in the context of joint picture book reading, children receive more ostensive input. Csibra and Gergely (2009) have suggested that infants are particularly sensitive toward ostensive cues. These cues signal to them that they are being addressed with information concerning generic knowledge. Thus, to receive ostensive input in a particular context—or domain (Gentner, 1988)—such as that of picture book reading, children are trained in applying cognitive operations such as categorizing objects (Gelman et al., 2005) or making relational comparisons (Rohlfing et al., 2015). This training, in turn, might facilitate the relational knowledge needed to understand spatial prepositions and in our study, this knowledge was rather already established by routines at homes more strongly than it could have been enhanced via experimental training.

However, although we found no learning effects with familiar objects in the task transfer, we did observe a marginal advantage of narrative condition for unfamiliar objects. We relate this observation to Son et al. (2012) who suggested that children show transfer abilities when more abstract examples are presented in the task. Following this idea, in the process of fading concreteness of memories via different tasks and less familiar objects, pictures can be beneficial for learning spatial prepositions, because the depicted events provide only a vague reference to the real world thus creating the need to add either the child’s or caregiver’s interpretation to the scene. In this way, more schematic forms of representations might emerge that contain the relational structure of objects (Son et al., 2012).

Further studies will need to disentangle memory demands resulting from a task and those resulting from the materials by systematically varying types of tasks and types of materials. For the moment, the present interpretation that picture books provide a useful pragmatic context in which semantically structured input in the form of narratives aids or strengthens a child’s lexical representations is limited to the method applied here. Our investigation indicates that successful encoding and retaining seem to be interrelated only when the encoding process is supported by semantic context in the input. There is therefore a need to consider intercontextual factors (such as semantic structure in the input and pragmatic task demands in the training and test) and intracontextual factors (children’s linguistic abilities) when investigating learning effects in children (Rohlfing et al., 2003).

## Author Contributions

KR supervised this study and wrote this paper, KN conducted the study. KR and KN analyzed the data.

## Conflict of Interest Statement

The authors declare that the research was conducted in the absence of any commercial or financial relationships that could be construed as a potential conflict of interest.
