# Supplementary material for: Can 28-Month-Old Children Learn Spatial Prepositions Robustly from Pictures? Yes, When Narrative Input Is Provided
Source: Front Psychol. 2016 Jul 15;7:961. doi: 10.3389/fpsyg.2016.00961 (PMC4945648; doi:10.3389/fpsyg.2016.00961)
Supplement: Supplementary file 1 [file Presentation_1.PDF]

Supplementary material

1. Language survey about children’s spatial words

Datum ______________

Fragebogen zu Sprache

Vorname des Kindes ____________________ ☐ weiblich ☐ männlich

Alter in Monaten ____________________

Geburtsdatum ____________________

Hat Ihr Kind Geschwister?

☐ ja

Alter: ____________________

Alter: ____________________

☐ nein

Adresse:

Name ____________________

Straße ____________________

PLZ/Ort ____________________

Bitte kreisen sie ein welche Wörter Ihr Kind versteht (👂) und welche es schon versteht und selbst spricht (👄):

**Handlungen**			passen (passt rein)	👂	👄	**Nomen**		
legen	👂	👄	auf(drehen)	👂	👄	Vorn	👂	👄
geben	👂	👄	zu(drehen)	👂	👄	Hinten	👂	👄
fallen	👂	👄	weg	👂	👄	Innen	👂	👄
verstecken	👂	👄	**Relationen zwischen Objekten**			Außen	👂	👄
hängen	👂	👄	Zu (auf etwas hin)	👂	👄	Oben (drauf)	👂	👄
drehen	👂	👄	Hinter	👂	👄	Unten	👂	👄
umdrehen	👂	👄	Vor	👂	👄	(auf der) Kante	👂	👄
hineintun (rein)	👂	👄	Zwischen	👂	👄	(in der) Mitte	👂	👄
herausnehmen (raus)	👂	👄	An (etwas dran)	👂	👄	Seite	👂	👄
öffnen (aufmachen)	👂	👄	In	👂	👄	Unterseite	👂	👄
schließen, (zumachen)	👂	👄	Auf	👂	👄	Loch	👂	👄
hoch	👂	👄	Unter	👂	👄			
runter	👂	👄	Über	👂	👄	**Andere**		
zusammen	👂	👄	Durch	👂	👄	hier!	👂	👄
auseinander	👂	👄	Um	👂	👄	wo?	👂	👄
an(schalten)	👂	👄	Bei	👂	👄	da!	👂	👄
aus(schalten)	👂	👄	Neben	👂	👄	dies / das	👂	👄

Kombiniert Ihr Kind schon zwei Wörter miteinander? (z.B. *Papa Arbeit!*), wenn ja, fallen Ihnen Beispiele dazu ein?

____

____

Dankeschön!

2. Stories for the training of the spatial preposition *behind*

Original German verbal input (in italics) with English translations (below)

Items	Experimental condition	Control condition
Rabbit, Cat, Hutch<br>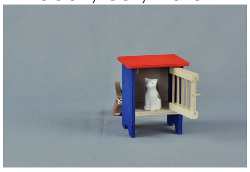	*Es ist ein sonniger Tag.*<br>*Der Hase möchte gerne seine Nachbarin besuchen.*<br>*Darum hüpft er zu der Katze und dem Stall.*<br>*Und wartet direkt hinter dem Stall, um sie zu überraschen.*<br><br>It's a sunny day.<br>The rabbit wants to visit his neighbor.<br>So he hops along to the cat and the hutch.<br>And waits directly behind the hutch to surprise her.	*Schauen wir uns das mal an!*<br>*Hier ist ein brauner Hase.*<br>*Und da ist noch eine graue Katze und das ist ein großer Stall.*<br>*Und der braune Hase steht hinter dem Stall.*<br><br>Let's have a look at this.<br>Here is a brown rabbit.<br>And there is a gray cat and this is a big hutch.<br>And the brown rabbit is behind the hutch.
Girl, Boy, Bench<br>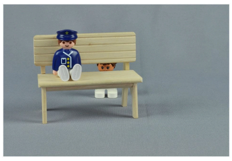	*Es ist nachmittags.*<br>*Das Mädchen und der Junge spielen verstecken.*<br>*Sie laufen zur Bank und der Junge hält sich die Augen zu.*<br>*Das Mädchen geht sofort hinter die Bank, damit er sie nicht finden kann.*<br><br>It is afternoon.<br>The girl and the boy are playing hide-and-seek.<br>They run to the bench and the boy closes his eyes.<br>The girl goes straight behind the bench, so that he cannot find her.	*Lass uns das mal kurz anschauen!*<br>*Hier ist ein heiteres Mädchen.*<br><br>*Und das ist ein netter Junge und da ist auch noch eine breite Bank.*<br>*Und das heitere Mädchen steht hinter der Bank.*<br><br>Let's take a quick look at this!<br>Here is a cheerful girl.<br><br>And this is a kind boy and there is also a wide bench.<br>And the cheerful girl is standing behind the bench.
Spoon, Tea bag, Cup<br>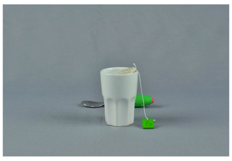	*Es ist früh morgens.*<br>*Jemand hat schon das Frühstück vorbereitet.*<br>*Der Löffel liegt weit weg von dem Teebeutel und dem Becher.*<br>*Man findet ihn aber hinter dem Becher und das Frühstück kann beginnen.*<br><br>It is early in the morning.<br>Someone has already prepared breakfast.<br>The spoon lies far away from the tea bag and the cup.<br>But you can find it behind the cup and breakfast can begin.	*Schauen wir uns dies mal an!*<br>*Hier ist ein grüner Löffel.*<br>*Und das ist ein leckerer Teebeutel und da ist auch noch ein runder Becher.*<br>*Und der grüne Löffel ist hinter dem Becher.*<br><br>Let's look at this.<br>Here is a green spoon.<br>And this is a tasty tea bag and there is also a round cup.<br>And the green spoon is behind the cup.
Umbrella, Hat, Wardrobe<br>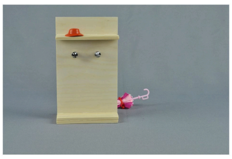	*Es ist sehr windig.*<br>*Der Schirm ist weg und wird als Schutz gesucht.*<br>*Er liegt nicht bei dem Hut und der Garderobe wie sonst immer.*<br>*Denn diesmal ist er hinter die Garderobe gefallen.*<br><br>It's very windy.<br>The umbrella is gone and we wanted it to keep us dry.<br>It is not in its usual place next to the hat and the wardrobe.<br>Because this time it fell down behind the wardrobe.	*Schau mal das hier!*<br>*Da ist ein rosa Schirm.*<br>*Und da ist noch ein brauner Hut und das ist eine Garderobe aus Holz.*<br>*Und der rosa Schirm ist hinter der Garderobe.*<br><br>Look at this!<br>There is a pink umbrella.<br>And there is also a brown hat and this is a wardrobe made of wood.<br>And the pink umbrella is behind the wardrobe.

3. Stories for the training of the spatial preposition next to

Original German verbal input (in italics) with English translations (below)

Items	Experimental condition	Control condition
Rabbit, Cat, Hutch<br>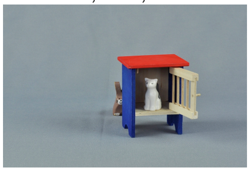	*Es ist ein warmer Tag.*<br>*Der Hase hat Hunger und würde gerne etwas essen.*<br>*Er sucht zuerst bei der Katze und dem Stall danach.*<br>*Und findet dann schließlich neben dem Stall was Frisches zu essen.*<br><br>It is a warm day.<br>The rabbit is hungry and wants to eat something.<br>It first looks near the cat and the hutch.<br>And finally finds something fresh to eat next to the hutch.	*Schau mal das hier!*<br>*Da ist ein junger Hase.*<br>*Und da ist auch noch eine alte Katze und das ist ein schöner Stall.*<br>*Und der junge Hase steht neben dem Stall.*<br><br>Look at this!<br>There is a young rabbit.<br>And there is also an old cat and this is a nice hutch.<br>And the young rabbit stands next to the hutch.
Girl, Boy, Bench<br>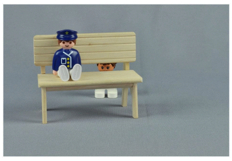	*Es sind Ferien.*<br>*Das Mädchen und der Junge wollen zusammen Eis essen gehen.*<br>*Sie sucht den Jungen und die Bank, um ihn abzuholen.*<br>*Schließlich steht sie neben der Bank und begrüßt ihn.*<br><br>It's vacation time.<br>The girl and the boy want to go an eat an ice cream together.<br>She looks for the boy and the bench in order to fetch him.<br>Finally, she stands next to the bench and says hello to him.	*Jetzt schauen wir uns das mal an!*<br>*Hier ist ein freundliches Mädchen und da ist ein fröhlicher Junge.*<br>*Und da ist noch eine lange Bank.*<br>*Und das freundliche Mädchen ist neben der Bank.*<br><br>Now, let's have a look at this!<br>Here is a friendly girl and there is a cheerful boy.<br>And there is a long bench.<br>And the friendly girl is next to the bench.
Spoon, Tea bag, Cup<br>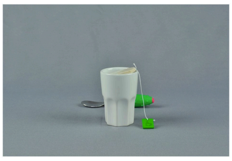	*Der Tee ist fast fertig.*<br>*Man braucht jetzt nur noch umzurühren.*<br>*Der Löffel liegt da, wo auch der Teebeutel und der Becher sind.*<br>*Er ist direkt neben dem Becher und man kann den Tee damit umrühren.*<br><br>Tea is almost ready.<br>It only needs to be stirred.<br>The spoon lies there, where the tea bag and the cup are, too.<br>It is directly next to the cup and you can stir the tea with it.	*Schauen wir uns das nächste mal kurz an!*<br>*Das ist ein kleiner Löffel.*<br>*Und hier ist ein loser Teebeutel und da ist ein weißer Becher.*<br>*Und der kleine Löffel ist neben dem Becher.*<br><br>Let's take a quick look at the next one!<br>This is a little spoon.<br>And here is a single tea bag and there is a white cup.<br>And the white spoon is next to the cup,
Umbrella, Hat, Wardrobe<br>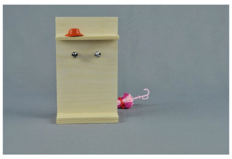	*Es ist schlechtes Wetter.*<br>*Der nasse Schirm ist aufgespannt und sehr groß.*<br>*Er passt jetzt nicht dahin, wo auch der Hut und die Garderobe sind.*<br>*Darum liegt er neben der Garderobe und kann dort gleich trocknen.*<br><br>The weather is bad.<br>The umbrella is wet and not folded up and is very big.<br>Now, it does not fit in where the hat and the wardrobe are.<br>That is why it is lying next to the wardrobe where it will soon be dry.	*Und dann gucken wir uns noch ein Bild an.*<br>*Hier ist ein bunter Schirm.*<br><br>*Und da ist noch ein dunkler Hut und das ist eine hohe Garderobe.*<br><br>*Und der bunte Schirm ist neben der Garderobe.*<br><br>Now let's look at another picture.<br>Here is a colorful umbrella.<br>And there is a dark hat and this is a tall wardrobe.<br>And the colorful umbrella is next to the wardrobe.
